# The role of vacant lots in promoting avian species diversity and occupancy in a post-industrial city

**DOI:** 10.1016/j.landurbplan.2025.105518

**Published:** 2025-10-08

**Authors:** Christopher J. Dennison, Amber L. Pearson, Jeffrey O. Hanson, Catherine D. Brown, Rachel T. Buxton

**Affiliations:** a Department of Biology and Integrated Science, Carleton University, Ottawa, Ontario, Canada; b Charles Stewart Mott Department of Public Health, Michigan State University, Flint, MI, USA; c Department of Geography, Environment and Spatial Sciences, Michigan State University, East Lansing, MI, USA

**Keywords:** Bird diversity, Bird occupancy, Green space, Urban biodiversity, Vacant land

## Abstract

In deindustrialized cities, human population decline and building demolitions have created large quantities of vacant land. Land vacancy is a complex social issue but may create habitat that supports wildlife. We explored the relationship between vacant land and other features of the urban landscape and bird diversity and occupancy in Detroit, Michigan. Acoustic recordings were collected annually in June at 110 sites across 11 Detroit neighbourhoods from 2021 to 2024. At each site we manually scanned 28 min of the morning chorus on two days to identify bird species. We compared resulting metrics of species diversity and occupancy with annual spatial data on neighbourhood characteristics including vacancy, vegetation, buildings, and roads measured in 50 m and 100 m buffers around each recording site. Using a mixed-effects modelling approach, we found higher bird species diversity and richness at recording sites surrounded by greater proportions of vacant land (Shannon diversity, β_vacantlots100m_ = 0.08, 95 % CI [0.04, 0.12]; bird species richness, IRR_vacantlots100m_ = 1.08, 95 % CI [1.04, 1.12]). Using a spatial occupancy modelling approach, we found vacant land was associated with higher occurrence of four bird species. However, other urban features, especially roads, were also associated with bird species diversity and occupancy. Our results suggest vacant land can support higher bird diversity and occupancy in Detroit neighbourhoods, while road infrastructure, buildings, and vegetation also play a role. These findings have implications for land management in Detroit and other deindustrialized cities, where there is increasing pressure to determine how vacant land can be used to achieve positive ecological and social outcomes.

## 1. Introduction

Urbanization is a major threat to biodiversity globally (Seto et al., 2012). Urban areas are characterized by higher densities of human-built infrastructure and roads which can destroy habitat, disrupt movement, and alter behaviour and communication by creating high levels of anthropogenic noise (Amaya-Espinel et al., 2019; Proppe et al., 2013). Due to these and other factors, cities are known to have a multitude of negative impacts on native birds (Isaksson, 2018). As such, there has been growing interest in understanding how urban areas can be designed to minimize negative impacts on birds and other forms of wildlife (Apfelbeck et al., 2020; Snep et al., 2015).

Although cities pose challenges to birds, they can also provide conditions that increase diversity and support rare or threatened species (Lepczyk et al., 2023). Human settlements have been established in landscapes that are important for biodiversity (Ives et al., 2016; Luck, 2007). Many cities are situated along key avian flyways and provide stopover or breeding sites for migratory species (La Sorte et al., 2020). Furthermore, while bird diversity generally decreases along gradients of urban development (Blair, 1996; Clergeau et al., 1998), several other factors can positively influence the occurrence and persistence of birds in cities. For example, the quantity and quality of green space and associated habitat is one of the major drivers of bird diversity and occurrence within and across urban areas (Beninde et al., 2015; Canedoli et al., 2018; Hughes et al., 2022). Well-managed city parks and even informal urban greenspaces, including residential yards and gardens, can support many different bird species and provide resources for habitat specialists (Aronson et al., 2017; Callaghan et al., 2018; Lepczyk et al., 2023; Villaseñor et al., 2020). However, given the unique structure and composition of cities, factors that affect birds are often city or location specific. Many cities exhibit atypical environmental characteristics that reflect complex socio-economic histories and processes, thus producing novel challenges for understanding and promoting urban biodiversity (Herrmann et al., 2016).

Deindustrialized cities, or those that have experienced human population decline and increasing vacancy due to loss of industry, are common in the American rust belt (a region of the northeastern and midwestern United States, as well as a small section of the southern United States) (Beauregard, 2009; Herrmann et al., 2016). Structurally, these cities are characterized by derelict infrastructure and buildings, and high quantities of vacant parcels of land (Hollander et al., 2009). While vacant lots often maintain some form of human-made structure, demolition and abandonment have made vacant land in many cities resemble disturbed open habitats with spontaneous, early successional vegetative communities comparable to meadows or grasslands (Johnson et al., 2018; Rega-Brodsky et al., 2018; Robinson & Lundholm, 2012; Tsuzuki et al., 2020). Vacant lots can also contribute to urban canopy cover by creating forest patches that support native and non-native trees (Riley et al., 2018; Zhang et al., 2024). Vacant land may support urban wildlife, including plants (Robinson & Lundholm, 2012), insects (Turo et al., 2021), mammals (Angold et al., 2006), and birds (Rega-Brodsky et al., 2018; Zuñiga-Palacios et al., 2020), some of which are of conservation concern (Meffert & Dziock, 2012; Schröder et al., 2018; Small et al., 2002). Although they create habitat and resources for wildlife, vacant lands are viewed negatively (e.g., as mismanaged or neglected areas used for crime) by some urban residents (Garvin et al., 2013; Rega-Brodsky et al., 2018; Sivak et al., 2021) and can have adverse effects on human health and wellbeing (Garvin et al., 2013; Pearson et al., 2021; Sivak et al., 2021).

There is currently a lack of research regarding conservation and biodiversity in deindustrialized cities like Detroit, despite the opportunities for innovative and sustainable use of vacant lands (Rega-Brodsky et al., 2018). Here we explored urban features that influence bird species diversity, richness, and occupancy in Detroit using acoustic recordings. We hypothesized that vacant land increases bird species richness, diversity and occurrence in Detroit neighbourhoods by creating diverse habitat and resources necessary for different birds to persist in an urban matrix (Gardiner et al., 2013; Horta et al., 2024). If vacant land supports birds by providing more diverse habitats, we should observe a positive association between bird diversity and richness at sites surrounded by more vacant land, as well as higher occupancy probability of birds with different habitat requirements. We discuss the implications of the relationship between birds, vacant land, and other urban features for revitalization strategies in Detroit.

## 2. Methods

### 2.1. Study area

Our study was based in Detroit, Michigan, USA, which has seen a decline in human population of more than one million residents since the 1950s (Desan, 2014). As a result, the city has high rates of land and building vacancy, with vacant land comprising approximately 17 % of total land coverage (~62 km^2^) across the city and more than 80 % in some neighbourhoods (Caverly, 2021; Peterson et al., 2020). Detroit also has historical and ongoing problems of social inequality and environmental racism which have resulted in marginalized communities being exposed to elevated levels of atmospheric pollutants and traffic noise (Shkembi et al., 2022). Many marginalized communities in Detroit also lack access to green space and the benefits these natural spaces afford (LaReaux & Watkins, 2025). We included 11 study neighbourhoods from across Detroit that were part of the broader Study of Active Neighborhoods Detroit (StAND) project, aimed at exploring the link between biodiversity, the rehabilitation of unmaintained green space, and human health (Pearson et al., 2020). There were no maintained or official parks in our study neighborhoods. To characterize bird communities, acoustic recordings were collected at 110 sites across 11 neighbourhoods for 1 week in June (ranging from June 2nd–June 25th) from 2021 to 2024 using AudioMoth v. 1.1.0 recorders (Hill et al., 2019) (Fig. 1).

### 2.2. Acoustic recordings

In each neighbourhood, one recorder was deployed at the centre of an unmanaged park and nine recorders were deployed at random locations within a 500 m grid. Recorders were separated by a minimum of 100 m (following Pearson et al., 2020). Although these recorders can detect loud species (e.g., Eurasian bittern, *Botaurus stellaris*) further than 100 m away in natural environments (Manzano-Rubio et al., 2022), we chose this particular minimum distance threshold because the transmission and detection of birdsong in cities are often degraded or masked by roads and anthropogenic noise (Nemeth & Brumm, 2010). Additionally, geophonic sounds (e.g., wind or rain) can also mask signal detection, further reducing the effective range of detection (Linares et al., 2020).

At each site, a recorder was placed in a plastic bag and attached to a tree or light pole at least 2 m above the ground. We recorded at a 48 kHz sampling rate with a gain setting of low (27.20 dB). Recorders were programmed to record for the first 5 min of each 10 min (six 5-minute recordings per hour) for the entirety of the 7-day recording period. Recorders were retrieved following the 7-day deployment period, and re-deployed if data was not collected or corrupted. Audio was saved in a. wav format. Technical and field complications with recorders (e.g., damaged or lost recorders and battery problems) led to several sites lacking acoustic data each year (2021*n* = 7; 2022*n* = 14; 2023*n* = 3; 2024*n* = 8). These sites were removed from analysis. All recorders were checked for functionality and firmware updates before deployment each year to ensure standardization across devices.

### 2.3. Acoustic analysis

To count and characterize bird sounds in acoustic recordings, we used Raven Pro v. 1.6.5 (K. Lisa Yang Center for Conservation Bioacoustics at the Cornell Lab of Ornithology, 2024) with presets: brightness: 50, contrast: 50, and resolution: 1050. For each recording site, on two randomly selected recording days, one observer conducted visual scans of spectrograms generated from the first 1-minute segment of each 5-minute recording from 5:50 am to 8:00 am and identified and recorded the presence of all avian species detected (14 1-minute segments per day; 28 1-minute segments per site, per year). Due to logistical constraints we used recordings from 6:00 am to 7:50 am in 2023. This method was based on recommended approaches to acoustic subsampling and analysis (Bayne et al., 2017; Hutschenreiter et al., 2024; Wimmer et al., 2013). Days that were randomly selected but had rain masking spectrograms were not used for analysis. For each 1-minute segment a bird was detected, it was marked with a “1″ (present) within that time segment. To identify unknown species sounds, we cross-referenced spectrograms with those of eBird (Sullivan et al., 2009) and used BirdNET, a machine learning-based model (Kahl et al., 2021). Unidentifiable sounds (less than 5 % of total sounds) were removed from analysis.

### 2.4. Spatial features at study sites

All spatial analyses were performed using ArcGIS Pro v. 3.3.1 software (Esri Inc., 2024). We mapped vacant lots in Detroit to examine their influence on avian communities. Here, we defined vacant lots as parcels of land without built structures or known public use (e.g., parking or agriculture). The structure and size of vacant land in Detroit is diverse, with some similar in structure to suburban lawns, remnant grasslands with shrubby edges, or even forest patches (Fig. 2).

Annual boundary files of vacant lots in study neighbourhoods were generated using publicly available datasets of building footprints and demolition data (Southeast Michigan Council of Governments (SEMCOG), 2023) and land parcel data (Urban, 2014), supplemented by in-person field audits. First, all land parcels not containing a built structure (*n* = 112,069) from the 2013–14 Motor City Mapping dataset were identified (Urban, 2014). Building footprint files from 2020 were then used to identify parcels which became vacant (i.e., contained no built structure) between 2013 and 2020 (*n* = 27,657). These parcels were merged with those extracted from the 2013–14 data to characterize all vacant lots in 2020 (*n* = 139,726). For subsequent years, building footprint data were used to determine which land parcels became vacant since the preceding year. These were cross-checked using Google Earth satellite images. Finally, we conducted in-person field audits from 2019 to 2023 to ensure accuracy.

To examine the influence of vegetation we obtained satellite imagery (1 m resolution) from the National Agriculture Imagery Program (NAIP) for the summers of 2018, 2020, and 2022 (National Agriculture Imagery Program − NAIP Hub Site, 2022). Although NAIP imagery for the state of Michigan was only available every second year, these data represent the best satellite imagery data available for the study area to our knowledge. Data for 2024 was not available for use as of November 2024. After aligning the NAIP imagery for each year to ensure consistency, we computed the Normalized Difference Vegetation Index (NDVI) to characterize spatial patterns of vegetation across the study area (Huang et al., 2021). As most urban parks in Detroit are unmaintained with spontaneous vegetation, NDVI also reflects the presence of green space around recording sites.

We extracted several other urban factors to investigate the effect of human-made structures. We used building footprints from 2021 and 2022 derived from boundary files developed by the Southeast Michigan Council of Governments (SEMCOG, 2024) and cross-referenced within our study area using Google Earth imagery and in-person audit data. A boundary file of impervious surfaces in Detroit was retrieved from the Detroit Water and Sewerage Department (Detroit Water and Sewerage Department (DWSD), 2022). This boundary file included measures of buildings, building remnants, decks and/or patio surfaces, driveways, pavement, parking lots, roads, sidewalks, and other impervious surfaces from 2022 (DWSD, 2022). We extracted road data from the DWSD impervious surfaces dataset to create a separate boundary file of roads for use in our analysis. In the absence of data for specific years, we used the most recent data available. We created 50 m and 100 m Euclidean buffers around each recording site for each year and calculated the percentage covered by vacant lots, buildings, roads, and impervious surfaces and average NDVI. These buffer sizes were selected to maximize independence between AudioMoth recorders based on the minimum 100 m distance between recorders and our estimated maximum detection distance of 100 m. These buffer sizes have also been shown to better capture information about diversity and vocal activity of birds relative to smaller (e.g., 25 m) or larger (e.g., 200 m) buffer sizes (Fairbairn et al., 2025).

### 2.5. Statistical analysis

All statistical analyses were performed using the R statistical computing environment (version 4.3.3; R Core Team, 2024). We assessed species diversity of birds at each site. Species diversity was evaluated using the Shannon-Weiner diversity (H’) metric (Oksanen et al., 2024) and species richness was calculated as the total number of unique species detected at each site. Because we were unable to distinguish individual birds, we used the proportion of 1-minute segments each species was detected during our manual count of birds over two random days as a proxy of relative abundance for calculating Shannon diversity (Fairbairn et al., 2025; Hutschenreiter et al., 2024). For example, if species *x* was detected in 14 of 28 1-minute segments analyzed at site *y* in 2021, proportion = 0.5 for this species. Although longer recording periods can reduce error in using acoustic metrics as proxies of relative abundance (Pérez-Granados et al., 2019), our high site number over four field seasons and use of manual rather than automated analysis of bird vocalizations necessitated a shorter analysis period to ensure efficiency.

We examined the effect of vacant lots and other environmental features on species diversity and richness using a mixed-effects modeling approach with a maximum likelihood estimation (Bates et al., 2015). We used a linear mixed-effects model to model Shannon diversity. For species richness, we used a generalized linear mixed-effects model and a Poisson distribution (Ver Hoef & Boveng, 2007). Preliminary visualization of dependent variables and fixed effects did not suggest a quadratic or cubic relationship. To accommodate the non-independence of observations from the same site and the same neighborhood, models were fitted with a site-level random intercept term nested within a neighborhood-level random intercept term. Year was also treated as a random effect to account for potential variability in avian vocal activity since our 7-day deployment periods (i.e., dates when AudioMoths were deployed) varied by year. However, we dropped year from our random effect structure in our global models for Shannon diversity and species richness, as a singular fit was observed due to this random effect (Pasch et al., 2013). Our global model for species richness also exhibited underdispersion, so we used Conway-Maxwell Poisson error structure (Brooks et al., 2017). To measure collinearity among environmental variables, we used Pearson’s correlation coefficient (Appendix A, Supplementary Data 1, Fig. S1). Variables with Pearson’s correlation coefficients above 0.6 were not included in the same model (Dormann et al., 2013; O’Brien, 2007). We centre-scaled all fixed effects to ensure resulting parameter estimates were comparable (Schielzeth, 2010).

We determined models best explaining Shannon diversity and species richness using an information theoretic approach (Bartoń, 2023; Mazerolle, 2006). In particular, we generated candidate models based on all possible combinations of fixed effects from the global model, excluding combinations that would result in candidate models containing collinear variables (i.e., variables with a correlation coefficient greater than 0.6). We then evaluated the parsimony of each candidate model using the Akaike Information Criterion corrected for small sample size (AICc) (Burnham & Anderson, 2002; Symonds & Moussalli, 2011) and calculated Akaike model weights (W_i_) (Wagenmakers & Farrell, 2004). Next, we evaluated the relative importance of each predictor variable (based on the sum of Akaike weights for models that included a given variable). We used a model averaging process to estimate model coefficients when more than one model exhibited support (ΔAICc < 2; Burnham & Anderson, 2002). When interpreting model coefficients, we assumed variables had a marginal effect if 95 % confidence intervals around parameter estimates did not include 0 for our linear mixed-effects models of Shannon diversity (Leroux, 2019). Similarly, we assumed variables had a marginal effect if 95 % confidence intervals around exponentiated incidence rate ratios (IRR) did not include one for our generalized linear mixed-effects models of species richness (Szumilas, 2010).

We performed additional analyses to verify model assumptions. We used residual plots to test model assumptions and to identify evidence of lack of fit in our global models (Hartig, 2022). We observed a slight deviation in the outlier test for our global model of Shannon diversity, but no other significant evidence of lack of model fit (Appendix A, Supplementary Data 1, Fig. S2). Residual plots for our global model of species richness models did not suggest lack of fit (Appendix A, Supplementary Data 1, Fig. S3). We calculated Nakagawa’s pseudo-R^2^ statistic to evaluate our best-fitting Shannon diversity and species richness models (Lüdecke et al., 2021; Nakagawa et al., 2017). We created a null model for Shannon diversity and species richness and compared its AICc to models exhibiting empirical support. Null models didn’t show empirical support (ΔAICc > 80). We tested for spatial autocorrelation in model residuals and verified that spatial autocorrelation was unlikely to bias inference (Hartig, 2022). Since sites were surveyed over multiple years, aggregated residuals derived from the same site were used to calculate Moran’s I for our global models (Hartig, 2022). We found no evidence of significant spatial autocorrelation in the Shannon diversity model (Global Moran’s I = − 0.03; *p* = 0.38) or species richness model (Global Moran’s I = 0.06; *p* = 0.10) (Appendix A, Supplementary Data 1, Table S1).

### 2.6. Occupancy models

We fitted occupancy models of bird species detected in recordings. We considered the two random days at each site from 2021 to 2024 as independent visits in our occupancy models, leading to two observations per year per site over four years. Species that were detected at a site between 5:50 am and 8:00 am on a randomly selected day were considered detected during that visit. We excluded species that were detected on less than 1 % of all days (*n* = 23), leaving 32 total species for analysis. To account for spatial autocorrelation in our detections, we used a spatial occupancy modelling approach (Doser et al., 2022). Spatial occupancy models use a Bayesian framework and allow for the inclusion of site-level spatial random effects to account for spatial autocorrelation (Doser et al., 2022). We included the proportion of land comprised of vacant lots in 50 m and 100 m buffers, proportion of land covered by buildings in 50 m and 100 m buffers, average NDVI in 50 m and 100 m buffers, and proportion of land covered by roads in 50 m and 100 m buffers as covariates affecting occupancy and Julian date for each visit as a covariate affecting detection. We included neighbourhood as an unstructured random effect in our occupancy models in addition to the site-level spatial random effect. Covariates with Pearson’s correlation coefficients above 0.6 were not included in the same model. A candidate set of stacked single-season spatial occupancy models for each species using detection data from 2021 to 2024 was created. Stacking combines repeat visit occupancy data over more than one year, with sites becoming site-year combinations accounted for with a site-level random effect (Ahlering et al., 2016; Bauder et al., 2017; Burnett & Roberts, 2015). We included a null spatial model with occurrence and detection covariates each set to one.

We fitted the occupancy models following best practices. In particular, we specified the spatial decay parameter as the mean distance between recording sites (Doser & Kéry, 2022). We used vague normal priors for variables affecting detection and occurrence and a vague inverse-Gamma prior for variance of our random effects (Doser & Kéry, 2022). An inverse-Gamma prior and uniform prior was used for spatial variance and our spatial range parameter, respectively (Doser & Kéry, 2022). We used an exponential spatial correlation function, assuming correlation between sites would decrease with increasing distance, and an autoregressive (AR(1)) temporal covariance framework to account for variation across years (Doser & Kéry, 2022). We used 1000 Markov Chain Monte Carlo (MCMC) batches of length 25 (*n-iterations* = 25,000) for six total chains in our spatial models (Doser & Kéry, 2022). Adequate MCMC convergence was assumed when Gelmin-Rubin statistic (Rˆ) values for all model parameters were below 1.1 (Gelman et al., 2013). In cases where Rˆvalues in our model of best-fit were above 1.1, we doubled our batch number to increase iterations until Rˆvalues were below 1.1.

After fitting the occupancy models, we used the Watanabe Akaike Information Criterion (WAIC) to evaluate their relative support (Watanabe, 2010). Since lower WAIC values indicate better support, we calculated a ΔWAIC value for each model – based on its WAIC relative to the best supported model – and considered models with ΔWAIC < 2 as competitive to the model of best fit (i.e., ΔWAIC = 0). This threshold is used often in information criterion-based model selection to indicate models with significant empirical support (Burnham & Anderson, 2002; Fay et al., 2021; Mészáros et al., 2025). In cases where more than one model was competitive, we used a Bayesian Model Averaging (BMA) approach to evaluate the importance of coefficients across all models showing support. BMA uses averaged posterior draws for parameters extracted from candidate models based on their weight, with higher weighted candidate models used for more draws (Bürkner, 2017; Hoeting et al., 1999). We used 95 % (equal-tailed) credible intervals for averaged occupancy coefficients to evaluate if covariates had a significant impact on occupancy (Makowski et al., 2019). We performed a posterior predictive check using the Freeman-Tukey test statistic and a Bayesian p-value to evaluate models of best-fit and all models that were competitive (Conn et al., 2018; Doser & Kéry, 2022) (Appendix A, Supplementary Data 2). We also compared the WAIC of our spatial model of best-fit and all models that were competitive to the WAIC of our null model to determine if our occurrence and/or detection covariates influenced the occupancy and detection of species.

## 3. Results

### 3.1. Species richness and diversity

A total of 54 species were detected across Detroit neighbourhoods in June from 2021 to 2024 (Appendix A, Supplementary Data 1, Table S2). Except for house sparrow (*Passer domesticus*) and European starling (*Sturnus vulgaris*), American robin (*Turdus migratorius*) was the most spatially and temporally ubiquitous species in our neighbourhoods (95.43 % of days analyzed), followed by Northern Cardinal (*Cardinalis cardinalis*; 82.35 % of days analyzed) and Chimney Swift (*Chaetura pelagica*; 70 % of days analyzed).

Vacant lots and roads in 100 m buffers showed the highest relative variable importance across all candidate models of Shannon diversity (Appendix A, Supplementary Data 1, Table S3). Our top performing model for Shannon diversity (ΔAICc = 0; W_i_ = 0.47) included vacant lots and roads within 100 m of recording sites and had marginal pseudo-R^2^ and conditional pseudo-R^2^ values of 0.37 and 0.52, respectively (Appendix A, Supplementary Data 1, Table S4). Three models exhibited empirical support (ΔAICc < 2). Vacant lots in 100 m buffers, roads in 100 m buffers, buildings in 50 m buffers and buildings in 100 m were present in at least one of the supported models Confidence intervals for model-averaged coefficients of buildings within 50 m and 100 m of recording sites included 0 (Appendix A, Supplementary Data 1, Table S5). There was a positive relationship between model-averaged proportion of vacant lots within 100 m of recording sites and Shannon diversity (β_vacantlots100m_ = 0.08, 95 % CI [0.04, 0.12]) and a negative relationship between model-averaged proportion of roads within 100 m of recording sites (β_roads100m_ = − 0.15, 95 % CI [− 0.18, − 0.11]) (Appendix A, Supplementary Data 1, Table S5; Fig. 3).

Vacant lots and roads in 100 m buffers showed the highest relative variable importance across all candidate models of bird species richness (Appendix A, Supplementary Data 1, Table S6). Three models exhibited empirical support (ΔAICc < 2). Vacant lots in 100 m buffers, roads in 100 m buffers, buildings in 50 m buffers and buildings in 100 m were present in at least one of the supported models (Appendix A, Supplementary Data 1, Table S7). Our model of best-fit for species richness ΔAICc = 0; W_i_ = 0.43) included buildings in a 50 m buffer and vacant lots and roads in 100 m buffers and had marginal pseudo-R^2^ and conditional pseudo-R^2^ values of 0.06 and 0.08, respectively. Confidence intervals for model-averaged coefficients (incidence rate ratios) of buildings within 50 m and 100 m of recording sites included 1 (Appendix A, Supplementary Data 1, Table S8). There was a positive relationship between model-averaged proportion of vacant lots within 100 m of recording sites and species richness (IRR_vacantlots100m_ = 1.08, 95 % CI [1.04, 1.12]) and a negative relationship between model-averaged proportion of roads within 100 m of recording sites (IRR_roads100m_ = 0.87, 95 % CI [0.85, 0.90]) (Appendix A, Supplementary Data 1, Table S8; Fig. 4).

### 3.2. Occupancy models

Vacant land had a positive association with the occupancy probability of four bird species. Eastern wood-pewee (*Contopus virens*; β_vacantlots50m_ = 2.02, 95 % CI [0.66, 3.63]) and northern cardinal (*Cardinalis cardinalis*; β_vacantlots50m_ = 1.36, 95 % CI [0.64, 2.13]) exhibited increased occupancy probability with higher proportions of vacant land within 50 m of recording sites while the occupancy probability of Baltimore oriole (*Icterus galbula*; β_vacantlots100m_ = 0.61, 95 % CI [0.11, 1.15]) and gray catbird (*Dumetella carolinensis*; β_vacantlots100m_ = 0.89, 95 % CI [0.47, 1.33]) increased with higher proportions of vacant lots within 100 m of recording sites (Fig. 5).

The proportion of buildings around recording sites had a negative effect on the occupancy probability of three bird species, and a positive effect on two species. The occupancy probability song sparrow (*Melospiza melodia*; β_buildings50m_ = − 1.34, 95 % CI [− 2.11, − 0.71]), warbling vireo (*Vireo gilvus*; β_buildings50m_ = − 1.56, 95 % CI [− 2.64, − 0.67]) decreased with higher proportion of buildings within 50 m. At the 100 m spatial scale, buildings decreased the occupancy probability of indigo bunting (*Passerina cyanea*; β_buildings100m_ = − 0.96, 95 % CI [− 1.55, − 0.38]). The occupancy probability of northern cardinal (β_buildings50m_ = 1.03, 95 % CI [0.36, 1.76]) and eastern wood-pewee (β_buildings100m_ = 1.51 95 % CI [0.05, 3.19]) increased with higher proportion of buildings in 50 m and 100 m buffers, respectively (Fig. 6).

Higher mean NDVI was associated with increased occupancy probability of one species at the 50 m spatial scale and three species at the 100 m spatial scale. Red-eyed vireo (*Vireo olivaceus*; β_NDVI50m_ = 0.92, 95 % CI [0.32, 1.65]) exhibited increased occupancy probability with higher mean NDVI in 50 m buffers while black-capped chickadee (*Poecile atricapillus*; β_NDVI100m_ = 0.93, 95 % CI [0.41, 1.53]), Carolina wren (*Thryothorus ludovicianus*; β_NDVI100m_ = 1.59, 95 % CI [0.75, 2.57]), and northern house wren (*Troglodytes aedon*; β_NDVI100m_ = 0.67, 95 % CI [0.31, 1.07]) had higher occupancy probability with higher mean NDVI in 100 m buffers (Fig. 6).

Proportion of roads in 100 m buffers had an exclusively negative effect on the occupancy probability of birds. Baltimore oriole (β_roads100m_ = − 0.55, 95 % CI [− 1.08, − 0.08]), downy woodpecker (*Picoides pubescens*; β_roads100m_ = − 0.81, 95 % CI [− 1.70, − 0.16]), gray catbird (β_roads100m_ = − 0.75, 95 % CI [− 1.22, − 0.34]), indigo bunting (β_roads100m_ = − 0.91, 95 % CI [− 1.48, − 0.39]), killdeer (*Charadrius vociferus*; β_roads100m_ = − 0.75, 95 % CI [− 1.34, − 0.24]), northern cardinal (β_roads100m_ = − 1.05, 95 % CI [− 1.60, − 0.58]), and song sparrow (β_roads100m_ = − 1.11, 95 % CI [− 1.73, − 0.57]) each had reduced probability of occupancy at sites with higher proportion of roads in 100 m buffers (Fig. 6).

## 4. Discussion

### 4.1. Shannon diversity and species richness

Our results show higher bird species diversity and richness at sites surrounded by higher proportions of vacant land and lower bird species diversity and richness at sites surrounded by more roads. Vacant land also exhibited a positive association with the occurrence of four songbirds, including one forest species. NDVI exhibited a positive association with four bird species and buildings exhibited a mix of positive and negative associations with bird occurrence. Roads exhibited an exclusively negative association with the occupancy probability of seven bird species.

Vacant land may support diverse bird communities by creating heterogeneous green space and habitat resources (e.g., insects, vegetation, nesting sites) in urban areas, where habitat is often limited (Anderson & Minor, 2019; Gardiner et al., 2013; Le Roux et al., 2014; Meffert and Dziock, 2012; Rega-Brodsky & Nilon, 2016). In Detroit and other cities, vacant lots vary widely in their structure, size, and composition, from suburban lawns with sparse vegetation to spontaneous grassland-meadows to small forest patches. As such, vacant land provides a diversity of habitat types that could benefit species with different habitat needs (Meffert and Dziock, 2012; Villaseñor et al., 2020; Zuñiga-Palacios et al., 2020). Interestingly, our most supported mixed-effects models for both bird diversity and richness included vacant land as a fixed effect and not NDVI, the latter being a proxy of primary productivity and vegetation density which has been shown to be associated with higher bird diversity (Fairbairn et al., 2025; Ju et al., 2024). At smaller spatial scales (e.g., 100 m), NDVI may be more sensitive to tree and grass cover relative to other vegetation types, such as shrubs and forbs (Martinez & Labib, 2023). As such, NDVI may represent a sufficient proxy of “greenness” more so than a direct measure of overall vegetation diversity, type, or complexity, especially in urban green spaces (Ju et al., 2024; Leveau & Isla, 2021). In contrast, vacant land may capture more information about the diverse habitat types and resources that support a greater diversity and richness of birds, and perhaps refugia for species less adapted to urbanization (Villaseñor et al., 2020).

We found evidence that roads had a negative association with bird diversity and species richness in Detroit. The negative impact of roads on birds is supported in the literature on road ecology (Cooke et al., 2020; Forman & Alexander, 1998). Highways and roads are a ubiquitous feature in cities and impact birds through fragmentation or destruction of habitat (Amaya-Espinel et al., 2019) and direct mortality via collisions with vehicles (Loss et al., 2014). Roads also create high levels of anthropogenic noise that degrade soundscape quality and disrupt behaviour and acoustic communication (Ware et al., 2015). Our results agree with previous studies, with roads exhibiting a negative association with species diversity and richness that was stronger than the positive association with vacant lots. Furthermore, since we measured roads as a proportion (i.e., percent coverage) around recording sites, our results may suggest that road type and density are important factors contributing to the avoidance of these structures by birds. This would support the argument that larger road types, such as multi-lane highways that support more traffic volume, have a stronger negative impact on birds relative to smaller roads (Cooke et al., 2020; Reijnen & Foppen, 2006). This effect, however, may not be generalizable across species, and would require further testing (Kroeger et al., 2022).

Although we found evidence of an association between species richness and the proportion of vacant lots and roads around recording sites, we found low goodness-of-fit (marginal pseudo-R^2^_=_ 0.06) compared to modelled Shannon diversity (marginal pseudo-R^2^_=_ 0.37). A possible explanation is that calculation of Nakagawa’s pseudo-R^2^ relies on distribution-specific variance (among others, Nakagawa et al., 2017), which may lead to unreliable results in models fit with a Conway-Maxwell Poisson error structure (Wiernik and Lüdecke, Personal Communication, August 28th, 2025). Another possible explanation is that vacant lots and roads capture more variation in Shannon diversity, a metric that reflects abundance and richness of species. Our use of the proportion of 1-minute segments in which a species was detected as a proxy for relative abundance may have also impacted model fit. For example, species that sing more often and for longer durations, such as American robin, could have inflated abundance estimates compared to species that only sing periodically and/or in short bursts. Furthermore, the low degree of variation in our species richness data, as evidenced by underdispersion, may explain poor model fit. With only a relatively small number of species occurring at recording sites, species richness may not be an optimal metric to explore ecologically relevant associations between birds and environmental factors in Detroit. Future studies could use point counts to complement passive acoustic monitoring for more accurate measures of relative abundance.

### 4.2. Bird occurrence

The results of our study suggest that some types of vacant land may benefit species with unique habitat requirements. For example, the occupancy probability of gray catbird, northern cardinal, and Baltimore oriole were positively associated with the proportion of vacant land around recording sites. These species depend on the availability of vegetated habitat to persist in cities. For gray catbird and northern cardinal, this includes dense, shrubby edge habitat, and for Baltimore oriole, mature deciduous trees (Balogh et al., 2011; Halkin et al., 2021; Nickell, 1965; Rising & Flood, 2020; Smith et al., 2020). In a previous study, Rega-Brodsky and Nilon (2016) found that the nest success of northern cardinal and gray catbird was higher in vacant lots with adequate densities of shrubby vegetation. We also found evidence that eastern wood-pewee occurrence increased with vacant land around recording sites. Eastern wood-pewee is an aerial insectivore that prefers deciduous forests and woodland clearings (Committee on the Status of Endangered Wildlife in Canada (COSEWIC), 2012; Falconer & Nol, 2021). Eastern wood-pewee is poorly adapted to urbanization, being found in lower densities in urban or peri urban environments relative to rural woodlands (Beissinger & Osborne, 1982). However, vacant land may provide trees and foraging opportunities to support this species. For example, vacant land can support insect diversity and abundance equal to or higher than gardens and surrounding urban areas, providing foraging opportunities for insectivores (Gardiner et al., 2013; Kattwinkel et al 2011). Although overall detection of eastern wood-pewee was low across our study (3.70 % of visits from 2021 to 2024), which may impact the robustness of this estimate, the association between eastern wood-pewee occurrence and vacant land could suggest that these spaces provide suitable habitat for specialist species.

We found a mix of positive and negative associations between other urban features and the occurrence of birds in Detroit. NDVI exhibited a positive association with the occupancy probability of four species. This included the red-eyed vireo, an insectivorous forest bird sensitive to urbanization (Chace & Walsh, 2006) that will persist in suburban areas where mature trees are present (Cimprich et al., 2020; Friesen et al., 1995). As such, higher NDVI may reflect increased canopy habitat for this forest bird at a local spatial scale. We also observed higher occupancy probability of Carolina wren, northern house wren, and black-capped chickadee with higher average NDVI. Although these generalists are likely more tolerant of urbanization than dietary or habitat specialists (Callaghan et al., 2019), association with greater average NDVI may reflect the need for cavity nesting sites and foraging opportunities (Foote et al., 2020; Haggerty et al., 2020; Johnson, 2024). Unlike NDVI, we found mostly negative associations between buildings and the occurrence of birds, including indigo bunting. Indigo buntings prefer well-vegetated edge habitats around open space (Bent & Austin et al., 1968; Johnston, 1965; Weldon & Haddad, 2005) and are less likely to occur in areas with higher building densities or in highly urbanized spaces (Burhans & Thompson, 2006; Payne, 2020). Interestingly, we observed a positive association between eastern wood-pewee and buildings at the 100 m scale, despite this species’ sensitivity to urbanization. However, this may reflect the low rate of detection for this species, leading to less reliable occupancy estimates. Lastly, we observed exclusively negative associations between roads around recording sites and the occupancy probability of birds, which supports previous studies suggesting a largely negative impact of roads and traffic on birds, especially small-bodied and/or migratory species (Cooke et al., 2020; Forman & Alexander, 1998).

The association between birds, NDVI, buildings, and roads in Detroit may further reflect the important role of vacant land for supporting urban bird populations. In large quantities, vacant land could create well-connected habitats unobstructed by buildings that provide habitat and promote movement between habitats (Anderson & Minor, 2017; Herbst & Herbst, 2006). In some Detroit neighbourhoods, vacant lots constitute more than 80 % of total land area, creating large swaths of open space and well-vegetated habitat. Given the negative impact of built structures on several species, vacant land could promote species sensitive to buildings (Villaseñor et al., 2020). For example, the negative influence of buildings on the occurrence of indigo bunting may be a reason for their relatively high rate of detection in Detroit (14.20 % of visits) despite their sensitivity to urbanization. Specifically, the propensity of this species to occur at sites surrounded by fewer buildings could indirectly reflect their preference for open areas, specifically those with more vacant land (Burhans & Thompson, 2006). Similarly, higher occurrence of several generalist and specialist bird species with greater NDVI may indicate a potential role for vacant land in supporting species which require suitable vegetative cover for nesting and foraging. The intentional improvement or rehabilitation of vacant lots (e.g., seeding or planting native vegetation) could strengthen the ability of these spaces to support birds (Anderson & Minor, 2017), including those that prefer habitats unobstructed by human-built structures and with sufficient vegetative cover, which may also provide refugia from hazardous aspects of the urban matrix, including roads.

### 4.3. Implications for vacant land management, urban greening, and planning

Our results must be considered within the diverse socio-ecological contexts that exist in Detroit, namely the negative effects of land vacancy and the legacies of inequality these spaces, and their future use, represent (Safransky, 2014). Management or improvement of vacant land in Detroit, including greening initiatives, must consider both the ecological potential of these spaces, such as increasing avian biodiversity, and also the social potential of these spaces to achieve holistic conservation outcomes (Phillips & Lindquist, 2021).

For example, research shows that the presence of vacant lots leads to feelings of unsafety and stress in local residents (Sivak et al., 2021). To manage its high percentage of vacant lands, the City of Detroit provides scheduled mowing (City of Detroit, 2024) to control vegetation growth and improve neighbourhood safety and perception of these spaces. Previous studies have shown resident preference for lots showing these signs of care or management and which have less emergent or dense vegetation (Rega-Brodsky et al., 2018). However, our results suggest a potentially high ecological value for unmaintained vacant lots for bird diversity, including perhaps lots with dense shrubby habitats and emergent vegetation that support birds like gray catbird and northern cardinal (Rega-Brodsky & Nilon, 2016). As such, mowing and removing vegetation may reduce biodiversity. Instead, we recommend that vacant land management in Detroit strive for a balanced approach that considers both the needs of residents and birds.

Numerous studies have shown the positive outcomes of greening initiatives that rehabilitate vacant lands, such as lowered feelings of stress and even reductions in gun violence (Branas et al., 2016; Branas et al., 2018; Sivak et al., 2021; South et al., 2018). As mentioned, however, improvements to vacant land that promote human wellbeing, such as mowing and reducing vegetation growth (i.e., reducing physical “disorder” and increasing “signs of care”), could reduce habitat resources and impact the ability of these spaces to support birds (Nassauer et al., 2021; Rega-Brodsky et al., 2018). Furthermore, increased human presence on managed or rehabilitated vacant lands may negatively impact birds sensitive to human activity compared to unused lots (Meffert & Dziock, 2012). As such, greening initiatives or management should be done considering socio-ecological factors and the need for balance between residents’ wellbeing and urban bird communities. For example, Detroit Bird City, a community-led program operated by Detroit Bird Alliance (formerly Audubon Detroit) and the City’s Parks and Recreation Department, rehabilitates vacant lots and unmanaged parks into walkable bird-meadow habitats (Phillips & Lindquist, 2021). Program staff and volunteers remove waste, invasive plants, and deteriorating impervious surfaces from these spaces, then seed native flowering plant communities to create bird and pollinator friendly habitats while maintaining surrounding shrubs and edge habitat. The installation of interpretive signage (e.g., bird identification boards), park benches, and pathways also invite use by neighbourhood residents and increased signs of care (Phillips & Lindquist, 2021). In a previous study of conservation of residential preferences for vacant lot management, Rega-Brodsky et al. (2018) found a majority of surveyed Baltimore residents preferred vacant land that maintained seating areas, trails, and flowering plants. Planning and management strategies that balance the needs and preferences of residents with the benefits vacant lots already provide birds should be prioritized to achieve the most sustainable and holistic socio-ecological outcomes for vacant land in Detroit.

In addition to the needs of local communities in Detroit, vacant land management should also consider the potential role of roads and their negative effect on birds. The diversity and abundance of birds in urban green space can be negatively affected by characteristics in the surrounding landscape, including high road density and resulting disturbance from traffic noise (Arévalo et al., 2022; Chamberlain et al., 2007; Cicort-Lucaciu et al., 2022; Melles et al., 2003). However, there is also evidence that birds will use green space and habitat structures in the presence of features in the surrounding landscape that may cause disturbance, as long as they are of sufficient size, density, or in proximity to other natural areas (Vasquez & Wood, 2022; Wood & Esaian, 2020). Regardless, management of vacant land should include the possibility that the conservation value of these spaces may be affected by aspects of the surrounding urban morphology (Arévalo et al., 2022). For example, vacant land rehabilitation in Detroit may be more successful in areas not surrounded by high densities of roads since roads appear to negatively impact the occupancy and diversity of birds in the city, likely due to physical disturbance and noise.

## 5. Conclusion

The results of our study have direct implications for urban conservation in Detroit and the sustainable and equitable use of vacant land in the city. Specifically, management and rehabilitation of vacant land for birds should consider how these spaces may already be providing habitat, and how aspects of the urban environment might impact the success of management and/or rehabilitation efforts. Furthermore, given that vacant land can have negative effects on people’s mental health, particularly marginalized residents, careful consideration for social justice and community consultation is needed to manage these spaces in a city still struggling with legacies of racism and segregation (Clement & Kanai, 2014; Foster & Newell, 2019; Stewart et al., 2019). Given the potential role vacant lands play in supporting bird diversity, current strategies of vacant land management, such as mowing, should be extended to include community-engaged reclamation of these spaces that achieves positive social outcomes while strengthening or maintaining their ecological role (Anderson & Minor, 2017; Kim et al., 2018; Schilling & Logan, 2008). Community-inclusive initiatives, such as Detroit Bird City, could strengthen the ability of vacant land to support birds while also enhancing the wellbeing of Detroit residents by increasing equitable access to nature, which has significant, positive health benefits (Buxton et al., 2021; Hartig et al., 2011; Keniger et al., 2013). The outcomes of these initiatives will require careful monitoring and future study to quantify their benefits to both people and birds (Pearson et al., 2020).

## Supplementary Material

1

2

## Figures and Tables

**Fig. 1.**
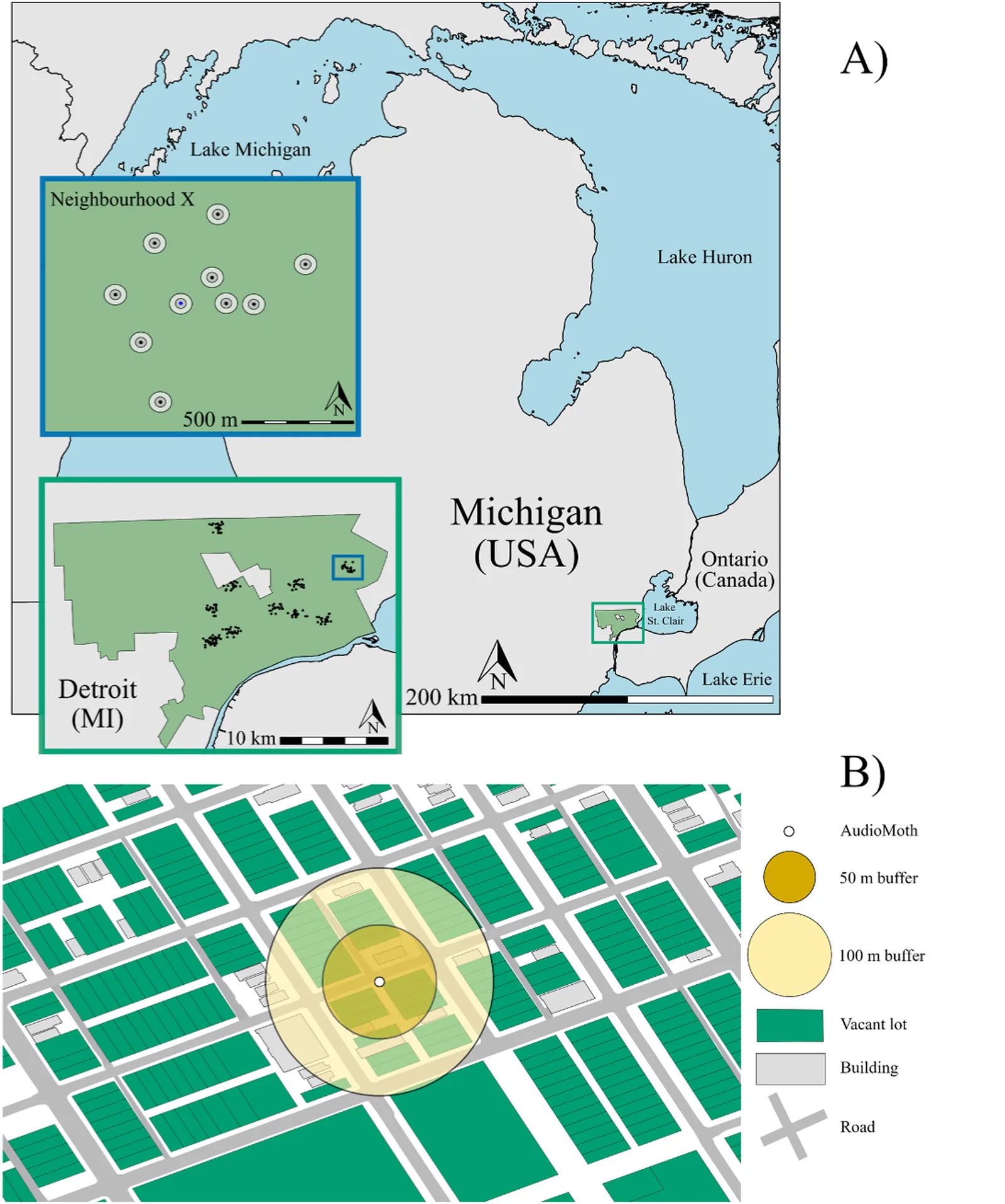
Location of Detroit in southeastern Michigan and location of all AudioMoth recording sites used from 2021 to 2024. “Neighbourhood X” provides an example arrangement of 10 AudioMoth recording sites (black dots − with 50 m and 100 m buffers shown around each site) in a Detroit neighbourhood (points jittered to protect site identity) **(A)**. Example recording location with surrounding urban features. Both 50 m and 100 m buffers are shown around the AudioMoth recorder **(B)**.

**Fig. 2.**
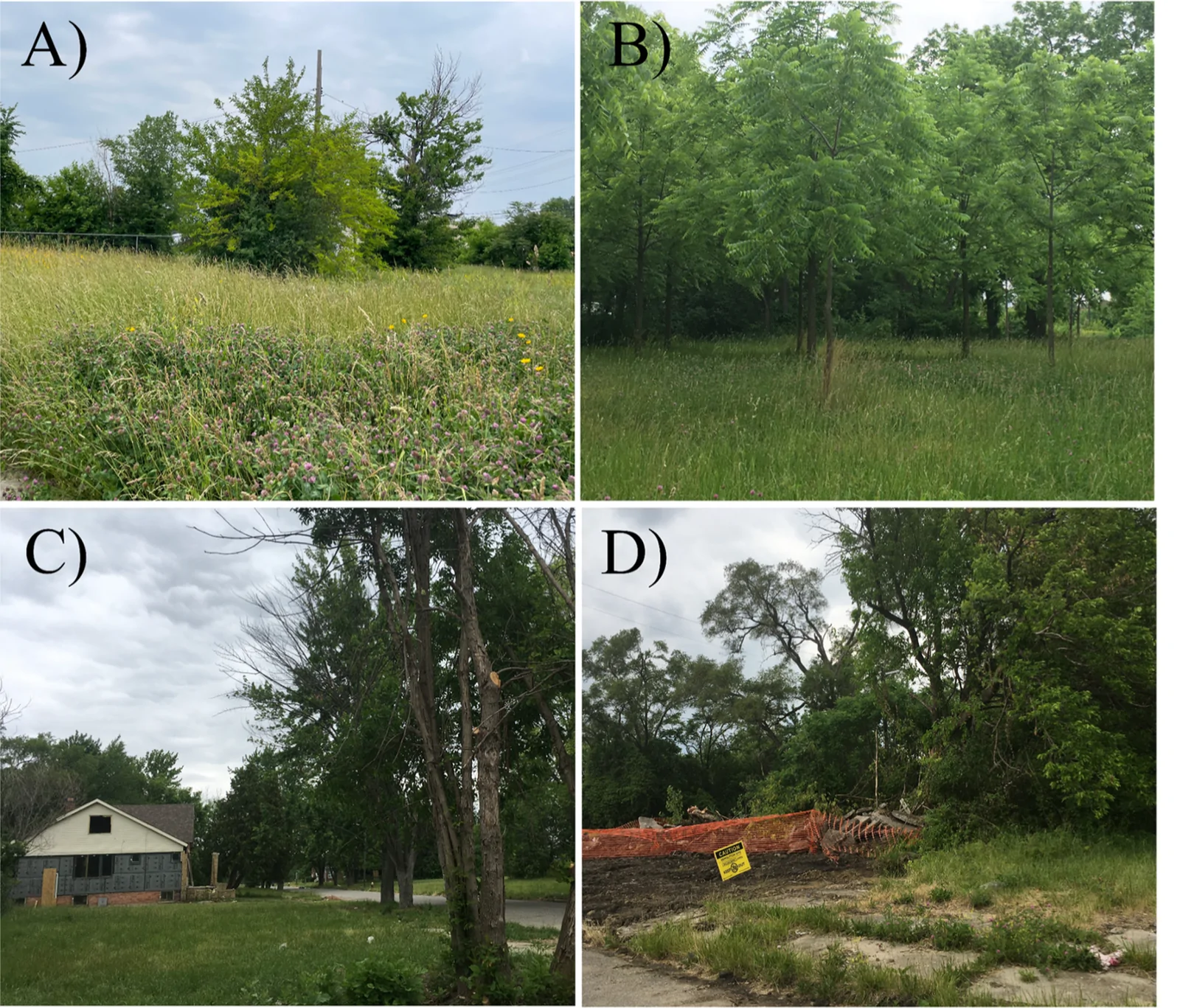
Example vacant lots from Detroit, MI. Vacant lots vary widely in structure and composition, and can include meadow-like habitats (**A**), forest patches (**B**), suburban lawns (**C**), and highly disturbed habitats (**D**).

**Fig. 3.**
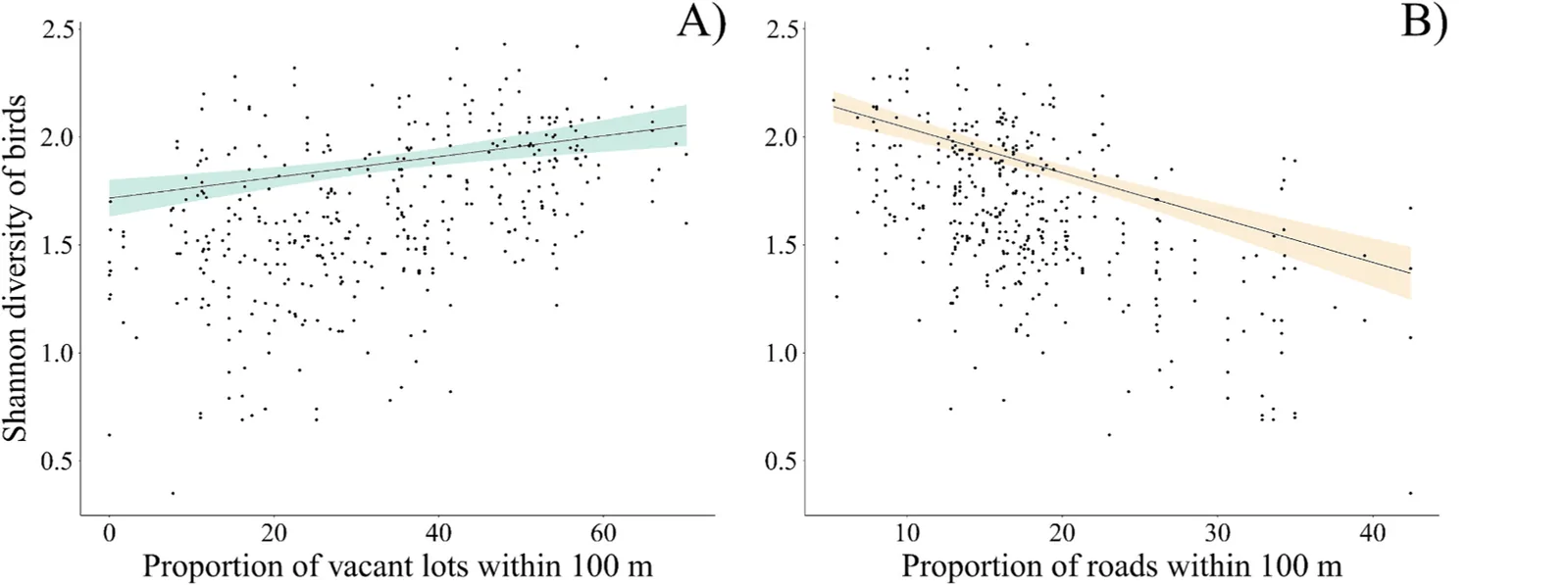
The effect of the proportion of vacant lots (**A**) and proportion of roads (**B**) in a 100 m buffer around each site on the average Shannon diversity of birds recorded at the sites around Detroit, MI. Lines correspond to predictions using coefficients from linear mixed-effects model of best-fit for Shannon diversity and silhouettes show 95 % prediction intervals. Marginal and conditional Nakagawa’s pseudo-R^2^ for model of best fit were 0.37 and 0.52, respectively.

**Fig. 4.**
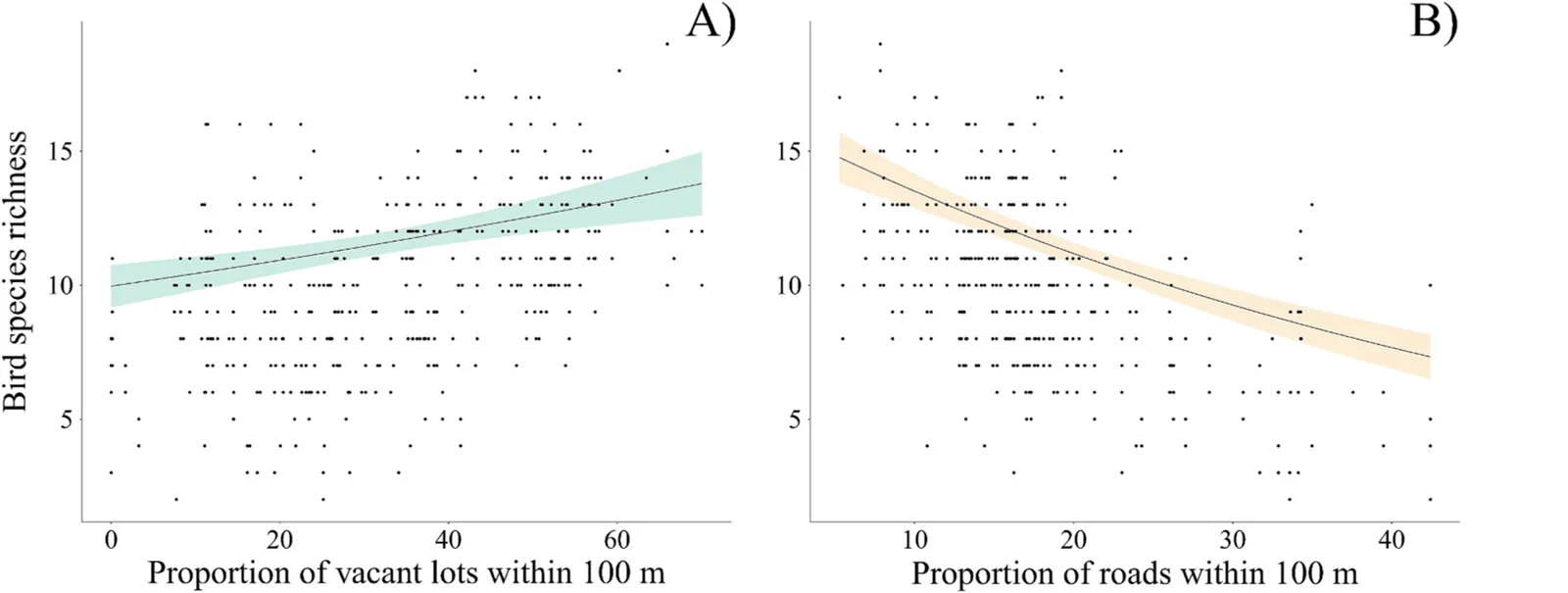
The effect of the proportion of vacant lots within 100 m of recording sites (**A**) and proportion of roads within 100 m of recording sites (**B**) on the species richness of birds recorded at the sites around Detroit, MI. Lines correspond to predictions of model-averaged coefficients from generalized linear mixed effects models (Conway-Maxwell Poisson) and silhouettes show 95% prediction intervals. Marginal and conditional Nakagawa’s pseudo-R^2^ for model of best fit were 0.06 and 0.08, respectively.

**Fig. 5.**
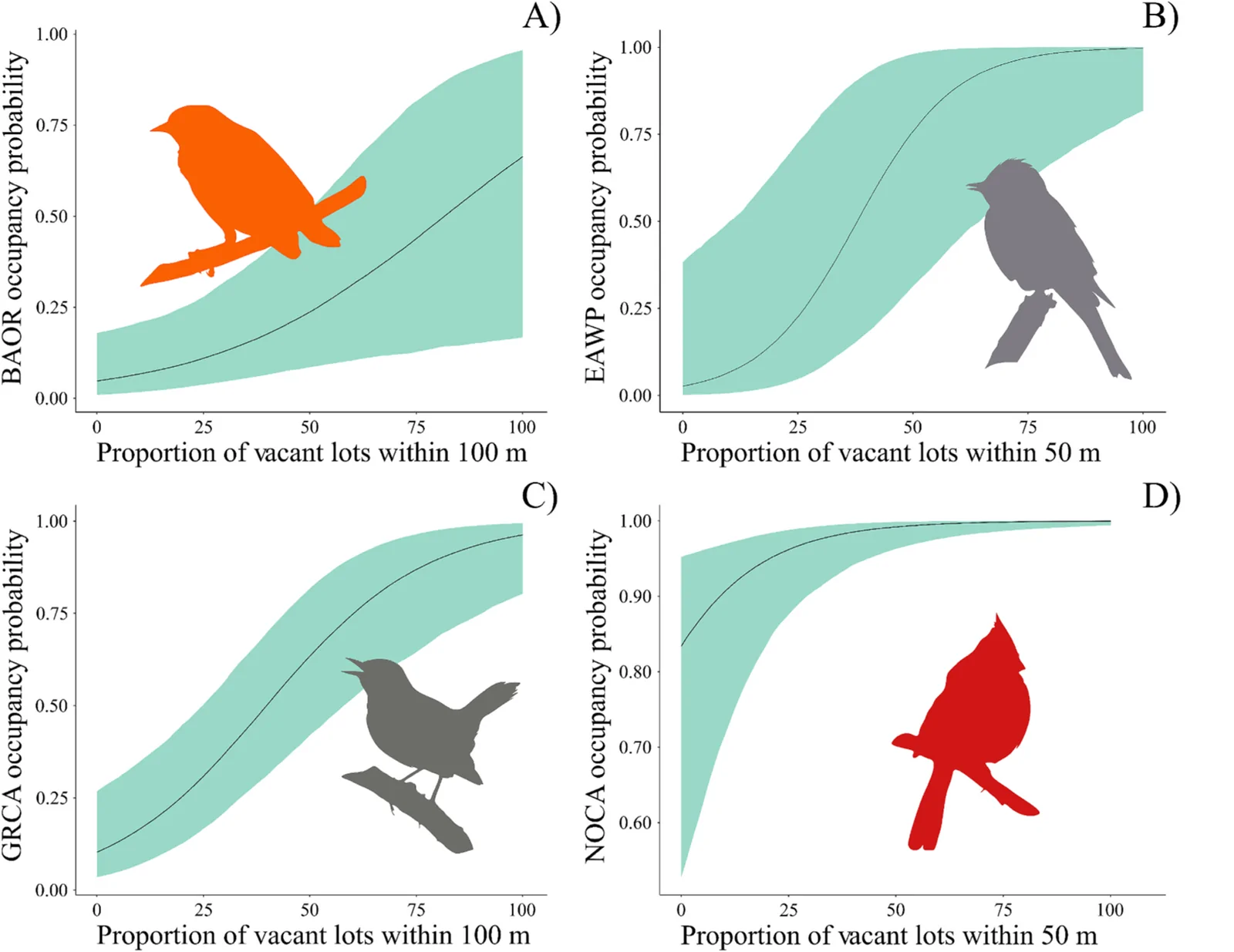
The effect of proportion of vacant lots within 50 m or 100 m of recording sites on the model-averaged occupancy probability of Baltimore oriole (BAOR) (**A**), eastern wood-pewee (EAWP) (**B**), gray catbird (GRCA) (**C**), and northern cardinal (NOCA) (**D**). Lines correspond to predictions of best-fitting spatial occupancy models, and silhouettes show 95 % prediction intervals.

**Fig. 6.**
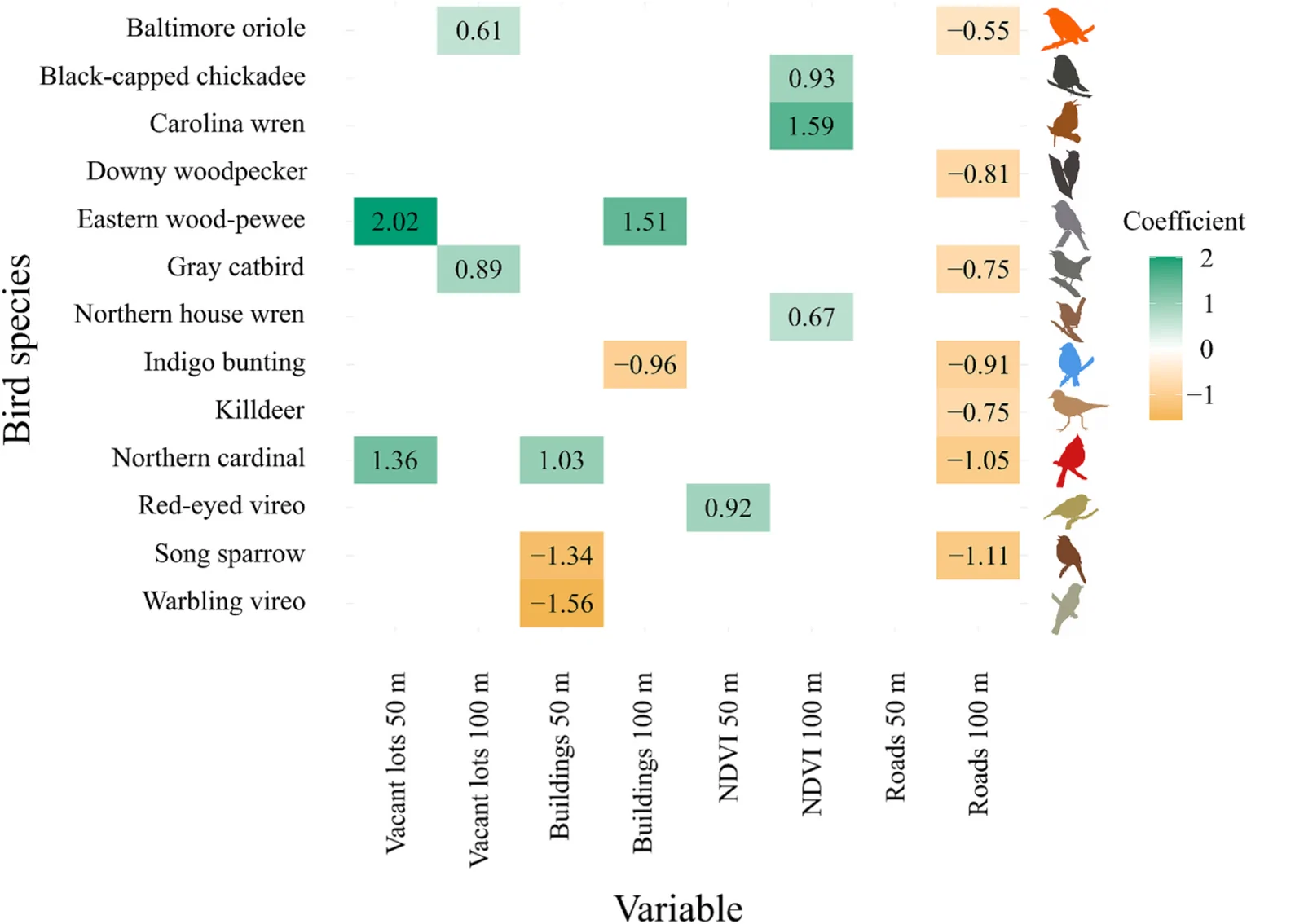
Association between predictor variables and the occupancy probability of bird species at recording sites in Detroit. Numbers represent model-averaged coefficients for variables whose 95 % credible intervals did not overlap 0.

## Data Availability

Data will be made available on request.

## H I G H L I G H T S

- Vacant land was associated with higher bird diversity in Detroit, while roads were associated with lower bird species diversity.
- The occurrence of birds with specific habitat requirements was higher in areas with more vacant land in Detroit.
- Other urban features, including roads, have an impact on the occurrence of birds in Detroit.
- Vacant land management and rehabilitation should consider biodiversity and promotion of social justice, and also the role of surrounding roads.

## Appendix A. Supplementary data

Supplementary data to this article can be found online at https://doi.org/10.1016/j.landurbplan.2025.105518.
