# Supplementary material for: The role of vacant lots in promoting avian species diversity and occupancy in a post-industrial city
Source: Landsc Urban Plan. Author manuscript; Available in PMC 2026 Oct 7. (PMC13641086; doi:10.1016/j.landurbplan.2025.105518)
Supplement: Supplementary file 1 [file NIHMS2184330-supplement-1.docx]

**Table S1** Results of Moran’s I test for spatial autocorrelation for linear mixed-effects models of best-fit for Shannon diversity and bird species richness.

|  | Shannon diversity | Species richness |
| --- | --- | --- |
| Expected Moran's I | −0.008 | −0.008 |
| Observed Moran's I | −0.03 | −0.06 |
| Standard deviation | 0.03 | 0.03 |
| p-value | 0.38 | 0.10 |

**Table S2** Birds detected in Detroit (2021–2024) sorted by percentage of visits (days) each species was detected.

| Species | Percent of visits detected |
| --- | --- |
| American Robin | 95.43 |
| European Starling | 91.23 |
| Northern Cardinal | 82.35 |
| House Sparrow | 81.11 |
| Chimney Swift | 70.00 |
| Brown-headed Cowbird | 55.80 |
| Ring-billed Gull | 52.22 |
| House Wren | 35.56 |
| Blue Jay | 35.43 |
| American Goldfinch | 33.21 |
| Common Grackle | 30.12 |
| Red-winged Blackbird | 30.12 |
| Gray Catbird | 26.30 |
| Ring-necked Pheasant | 26.17 |
| Northern Flicker | 19.38 |
| Downy Woodpecker | 18.77 |
| Black-capped Chickadee | 17.41 |
| Song Sparrow | 16.30 |
| Chipping Sparrow | 14.32 |
| Indigo Bunting | 14.20 |
| Baltimore Oriole | 11.60 |
| Mourning Dove | 10.37 |
| Killdeer | 10.12 |
| American Crow | 7.90 |
| Warbling Vireo | 4.69 |
| House Finch | 4.32 |
| Great Crested Flycatcher | 4.32 |
| Common Nighthawk | 4.20 |
| Red-eyed Vireo | 3.83 |
| Eastern Wood-Pewee | 3.70 |
| Yellow Warbler | 3.21 |
| Carolina Wren | 3.09 |
| Eastern Kingbird | 1.23 |
| Hairy Woodpecker | 0.99 |
| Common Yellowthroat | 0.99 |
| American Kestrel | 0.74 |
| White-breasted Nuthatch | 0.62 |
| Orchard Oriole | 0.49 |
| Willow Flycatcher | 0.37 |
| American Redstart | 0.37 |
| Eastern Meadowlark | 0.37 |
| Cedar Waxwing | 0.25 |
| Eastern Phoebe | 0.25 |
| Black-and-white Warbler | 0.25 |
| Barn Swallow | 0.12 |
| Blue-gray Gnatcatcher | 0.12 |
| Acadian Flycatcher | 0.12 |
| Ovenbird | 0.12 |
| Swainson's Thrush | 0.12 |
| Tennessee Warbler | 0.12 |
| Nashville Warbler | 0.12 |
| Black-throated Green Warbler | 0.12 |
| Hermit Thrush | 0.12 |
| Blackpoll Warbler | 0.12 |

**Table S3** Sum of model weights including predictor variables across all candidate linear mixed-effects models for Shannon diversity.

| Variable | Sum of model weights |
| --- | --- |
| Roads (100 m) | 1.00 |
| Vacant Lots (100 m) | 9.77×10^-01^ |
| Buildings (50 m) | 2.88×10^-01^ |
| Buildings (100 m) | 2.45×10^-01^ |
| NDVI (100 m) | 1.48×10^-02^ |
| Vacant Lots (50 m) | 6.64×10^-03^ |
| NDVI (50 m) | 1.92×10^-03^ |
| Roads (50 m) | 1.03×10^-10^ |

**Table S4** Model selection (AICc) table for Shannon diversity linear mixed-effects models. Model of best-fit (W_i_ = 0.47) includes proportion of vacant lots and roads within 100 m of recording sites.

| (Intercept) | Buildings (100 m) | Buildings (50 m) | NDVI  (100 m) | NDVI  (50 m) | Roads (100 m) | Roads (50 m) | Vacant lots  (100 m) | Vacant lots  (50 m) | Df | LogLik | AICc | Δ AICc | w_i_ |
| --- | --- | --- | --- | --- | --- | --- | --- | --- | --- | --- | --- | --- | --- |
| 1.87 | NA | NA | NA | NA | −0.15 | NA | 0.08 | NA | 6 | −6.33 | 24.87 | 0.00 | 0.47 |
| 1.87 | NA | −0.02 | NA | NA | −0.15 | NA | 0.07 | NA | 7 | −5.80 | 25.89 | 1.02 | 0.28 |
| 1.87 | −0.02 | NA | NA | NA | −0.15 | NA | 0.07 | NA | 7 | −6.00 | 26.28 | 1.41 | 0.23 |
| 1.87 | −0.05 | NA | 0.04 | NA | −0.16 | NA | NA | NA | 7 | −9.61 | 33.50 | 8.63 | 6.25×10^−03^ |
| 1.87 | NA | −0.04 | 0.04 | NA | −0.16 | NA | NA | NA | 7 | −10.07 | 34.42 | 9.54 | 3.95×10^−03^ |
| 1.87 | −0.05 | NA | 0.04 | NA | −0.16 | NA | NA | 0.006 | 8 | −9.55 | 35.47 | 10.60 | 2.33×10^−03^ |
| 1.87 | −0.06 | NA | NA | NA | −0.18 | NA | NA | NA | 6 | −11.65 | 35.50 | 10.63 | 2.29×10^−03^ |
| 1.87 | −0.05 | NA | NA | NA | −0.17 | NA | NA | 0.02 | 7 | −10.93 | 36.15 | 11.28 | 1.66×10^−03^ |
| 1.87 | NA | −0.04 | 0.04 | NA | −0.16 | NA | NA | 0.004 | 8 | −10.04 | 36.45 | 11.58 | 1.43×10^−03^ |
| 1.87 | −0.05 | NA | NA | 0.02 | −0.17 | NA | NA | NA | 7 | −11.12 | 36.52 | 11.65 | 1.38×10^−03^ |

| Variable | Beta | 95% CI*^1^* |
| --- | --- | --- |
| Buildings (50 m) | −5.61×10^−03^ | −0.06, 0.02 |
| Buildings (100 m) | −4.15×10^−03^ | −0.06, 0.02 |
| **Vacant Lots (100 m)** | **0.08** | **0.04, 0.12** |
| **Roads (100 m)** | **−0.15** | **−0.18, −0.11** |
| *^1^*Confidence interval | | |

**Table S5** Model-averaged parameter values from supported (ΔAICc <2) linear mixed-effects models of bird Shannon diversity. Bolded text indicates 95% confidence intervals not overlapping 0.

**Table S6** Sum of model weights including predictor variables across all candidate generalized linear mixed-effects models for species richness.

| Variable | Sum of model weights |
| --- | --- |
| Roads (100 m) | 1.00 |
| Vacant Lots (100 m) | 9.77×10^-01^ |
| Buildings (50 m) | 4.38×10^-01^ |
| Buildings (100 m) | 2.06×10^-01^ |
| NDVI (100 m) | 1.98×10^-02^ |
| Vacant Lots (50 m) | 8.72×10^-03^ |
| NDVI (50 m) | 9.08×10^-04^ |
| Roads (50 m) | 1.42×10^-10^ |

**Table S7** Model selection (AICc) table for bird species richness generalized linear mixed-effects models. Model of best-fit (W_i_ = 0.43) includes proportion of buildings within 50 m of recording sites and proportion of vacant lots and roads within 100 m of recording sites.

| (Intercept) | Buildings 100 m | Buildings 50 m | NDVI 100 m | NDVI 50 m | Roads 100 m | Roads 50 m | Vacant lots 100 m | Vacant lots 50 m | Df | LogLik | AICc | Δ AICc | w_i_ |
| --- | --- | --- | --- | --- | --- | --- | --- | --- | --- | --- | --- | --- | --- |
| 2.45 | NA | −0.03 | NA | NA | −0.14 | NA | 0.07 | NA | 7 | −954.90 | 1,924.09 | 0.00 | 0.43 |
| 2.45 | NA | NA | NA | NA | −0.13 | NA | 0.08 | NA | 6 | −956.12 | 1,924.46 | 0.37 | 0.36 |
| 2.45 | −0.02 | NA | NA | NA | −0.14 | NA | 0.07 | NA | 7 | −955.68 | 1,925.65 | 1.56 | 0.20 |
| 2.45 | NA | −0.05 | 0.05 | NA | −0.14 | NA | NA | NA | 7 | −958.99 | 1,932.27 | 8.18 | 7.15×10^−03^ |
| 2.45 | −0.05 | NA | 0.05 | NA | −0.14 | NA | NA | NA | 7 | −959.23 | 1,932.75 | 8.66 | 5.62×10^−03^ |
| 2.45 | NA | −0.04 | 0.04 | NA | −0.14 | NA | NA | 0.01 | 8 | −958.73 | 1,933.83 | 9.73 | 3.28×10^−03^ |
| 2.45 | −0.04 | NA | 0.04 | NA | −0.14 | NA | NA | 0.02 | 8 | −958.83 | 1,934.03 | 9.94 | 2.96×10^−03^ |
| 2.45 | −0.05 | NA | NA | NA | −0.16 | NA | NA | 0.03 | 7 | −960.88 | 1,936.05 | 11.96 | 1.08×10^−03^ |
| 2.45 | NA | −0.04 | NA | NA | −0.15 | NA | NA | 0.03 | 7 | −961.08 | 1,936.45 | 12.36 | 8.83×10^−04^ |
| 2.45 | −0.05 | NA | NA | 0.03 | −0.15 | NA | NA | NA | 7 | −961.49 | 1,937.26 | 13.17 | 5.89×10^−04^ |

**Table S8** Model-averaged parameter values from supported (ΔAICc <2) generalized linear mixed-effects models of bird species richness. Bolded text indicates 95% confidence intervals not overlapping 1.

| Variable | IRR*^1^* | 95% CI | |
| --- | --- | --- | --- |
| **Vacant Lots (100 m) mmm)** | **1.08** | **1.04,** **1.12** | |
| Buildings (50 m) | 0.99 | 0.94, 1.00 | |
| Buildings (100 m) | 1.00 | 0.95, 1.02 | |
| **Roads (100 m)** | **0.87** | **0.85, 0.90** | |
| *^1^* Incidence rate ratio (exponentiated) | | |  |

## **Supplementary Figures**

**Figure S1** Pearson’s correlation matrix for fixed effects using in mixed-effects models.

**Figure S2** QQplot and observed vs. predicted plots of model residuals for global Shannon diversity linear mixed effects model. Created with the *DHARMa* package (Hartig, 2022).

**Figure S3** QQplot and observed vs. predicted plots of model residuals for global bird species richness generalized linear mixed effects model (Conway-Maxwell) Created with the *DHARMa* package (Hartig, 2022).
